# Navigating Scientific Inquiry in East Africa Amidst Declining Research Funding

**DOI:** 10.24248/eahrj.v9i1.816

**Published:** 2025-09-30

**Authors:** Steve Wandiga, Leonard Ntakarutimana, Fabian Mashauri, Novat Twungubumwe

**Affiliations:** a Kenya Medical Research Institute; b East African Health Research Commission

**Keywords:** East Africa, research funding crisis, sustainable research models, scientific collaboration, resource optimization, community-based research, diversified funding strategies, methodological innovation, South-South partnerships, research capacity building.

## Abstract

East African scientific research confronts an unprecedented funding crisis that has fundamentally transformed the region's research landscape. Traditional funding mechanisms, heavily dependent on international grants and donor aid, have proven increasingly unsustainable amid global economic volatility, shifting donor priorities, and persistent COVID-19 impacts. Between 2022 and 2024, research funding to sub-Saharan Africa declined by approximately 18%, with East Africa experiencing particularly severe reductions as university research budgets stagnated while inflation eroded purchasing power by 15–20% annually across Kenya, Uganda, Tanzania, Rwanda, Ethiopia, and Sudan.

Even if the traditional funding reduction undermined scientific production in the EA region at the beginning, an impressive capacity of resiliency and innovation was thereafter quickly observed that catalyzed unprecedented innovation and collaboration among researchers. Rather than diminishing scientific capacity, this funding crisis has catalyzed unprecedented innovation and collaboration, spurring the development of resilient, self-sustaining research models that maintain scientific excellence despite severe resource constraints. This paper examines 6 transformative case studies that demonstrate how East African researchers have revolutionized scientific inquiry approaches, creating sustainable alternatives to traditional funding-dependent models through a comprehensive analysis spanning 2020–2025.

The cases described in this paper reveal comprehensive strategic frameworks integrating diversified funding portfolios, resource-efficient methodologies, robust community engagement ensuring research relevance, and collaborative networks facilitating expertise and infrastructure sharing. Successful programs strategically leverage expanding digital connectivity, open science practices, and South-South partnerships while systematically building sustainable local capacity and maintaining rigorous scientific standards. Strategic policy engagement and research translation create additional sustainability pathways by aligning research priorities with pressing societal needs and generating diverse funding opportunities.

## BACKGROUND

East Africa's scientific research landscape is facing unprecedented challenges as traditional funding sources are diminishing while regional research needs are intensifying. The progressive decrease of funds availability from 2020 to 2025 has significantly impacted research activities across the region. The COVID-19 pandemic negatively impacted research funds availability starting from the first cases in East African countries in 2020, with effects continuing through 2024. This progressive funding decline resulted in reduced research activities, limited researcher mobility, decreased international collaboration, and postponed or cancelled research projects across the region during this critical period. The COVID-19 pandemic, shifting donor priorities, and persistent economic pressures have created a perfect storm of reduced funding availability, forcing researchers across Kenya, Uganda, Tanzania, Rwanda, Ethiopia, and Sudan to rethink how they conduct scientific inquiry fundamentally.

Yet within this challenging context, East African researchers have demonstrated remarkable resilience and innovation, developing strategies that offer valuable lessons for navigating scientific inquiry under severe resource constraints. This communication examines proven approaches, successful case studies, and emerging opportunities that enable continued scientific excellence despite financial limitations.

The Eastern Africa Consortium for Clinical Research exemplifies sustained excellence through diversified funding strategies and adaptive collaboration, operating successfully for over 15 years while training 33 master's students and conducting multi-country studies at 50% lower per-participant costs than single-country equivalents. Makerere University's climate-health program transformed a devastating 65% budget reduction into innovative opportunity, developing community-based participatory research that reduced data collection expenses by 85% while generating unprecedented policy impact and attracting subsequent funding. The East African Bioinformatics Resource Consortium demonstrates strategic resource pooling's transformative potential, enabling world-class analytical capabilities at fractional individual institutional costs while achieving 70% operational sustainability through diversified service offerings.

This transformation demonstrates that resource constraints, while initially challenging, can drive methodological innovation and collaborative approaches that ultimately strengthen research programs beyond what traditional well-funded models achieve. East African researchers implementing these approaches position themselves not merely to survive challenging funding environments but to develop more sustainable, impactful, and community-relevant research programs that contribute meaningfully to regional development priorities and global scientific advancement.

## METHODS

This study employed a scoping review methodology to systematically examine and synthesize documented sustainable research strategies in East Africa during the 2020–2025 funding crisis period. The approach focused on mapping the existing evidence and identifying key characteristics and outcomes of adaptive research models.

### Case Identification and Selection

Six representative cases were identified and selected for in-depth examination based on the following criteria: (1) documented evidence of maintaining research operations through funding reductions of ≥50%, (2) operational continuity for a minimum of 3 years during the study period, (3) clear documentation of innovative adaptation strategies, (4) geographic representation across East African countries, and (5) diversity across research domains including clinical research, environmental health, bioinformatics, digital health, surveillance, and agriculture.

### Data Collection and Sources

Data were gathered through comprehensive document analysis of publicly available and institutional sources including:

- Peer-reviewed publications and preprints documenting case outcomes
- Institutional annual reports, strategic plans, and public statements
- Grant reports and funding announcements
- Financial statements and budget documentation where accessible
- Policy documents and white papers from national and regional science agencies
- Conference presentations and proceedings related to the cases

### Analytical Approach

The analysis followed established scoping review frameworks to map the key characteristics, strategies, and outcomes of each case. Document analysis focused on identifying patterns of adaptation, financial sustainability indicators including funding diversification approaches and cost reduction methodologies, and common challenges across cases. Comparative analysis was conducted to identify cross-cutting themes and distinctive approaches across different research domains and national contexts.

Validation was achieved through triangulation across multiple documentary sources and cross-verification of reported data points where possible. The synthesis aimed to provide a comprehensive overview of documented strategies and outcomes without making causal claims about effectiveness.

### The East African Research Funding Crisis

The African continent, and particularly sub-Saharan Africa, has experienced significant challenges in research funding over recent years. This broader continental crisis has manifested most acutely in East Africa, where countries typically depend heavily on external funding for research activities. East African countries typically depend heavily on external funding for research activities, with domestic research and development spending averaging less than 0.3% of GDP across the region^1^. This dependency creates acute vulnerability when international funding priorities shift or economic downturns reduce donor capacity or willingness. The success of research in East Africa needs public, private, and academic sector partnerships to secure self-funding and control over agendas and priorities, highlighting the critical need for sustainable funding models.

Recent analyses indicate that research funding to sub-Saharan Africa decreased by approximately 18% between 2022 and 2024, with East Africa experiencing some of the steepest declines^2^. The situation is particularly acute in countries like Uganda and Tanzania, where university research budgets have remained stagnant while inflation erodes purchasing power by an estimated 15 to 20% annually.^3^

Despite challenges like climate events, conflicts, high debt, and depreciating currency value, East Africa remains the fastest-growing region in Africa with GDP growth rates averaging 4.5 to 5.2% annually from 2020–2024^4^, suggesting potential for innovative funding solutions that align research priorities with regional development needs.

### Case Study 1: The Eastern Africa Consortium for Clinical Research - A Model of Sustained Excellence

The Eastern Africa Consortium for Clinical Research (EACCR) is an Eastern African-led network established in 2009 and comprises 23 regional partners from Ethiopia, Kenya, Rwanda, Sudan, Tanzania, and Uganda, and 5 Northern partners from Belgium, the Netherlands, Norway, Sweden, and the United Kingdom. After operating for over 15 years, the EACCR represents the most successful example of sustained collaborative clinical research in the region^5,6^, demonstrating remarkable resilience through multiple funding cycles and economic upheavals.

EACCR has carried out multiple laboratory upgrades including 15 molecular biology laboratories, 8 clinical chemistry facilities, and 12 microbiology units across underdeveloped network sites. In total, 33 master's students, five PhD students, and five postdoctoral researchers have benefited from long-term training, showcasing how strategic capacity building creates sustainable research infrastructure that transcends individual funding cycles. During the last 5 years, 18 Master's, 3 PhD, and 2 postdoctoral researchers were trained using a distributed mentorship strategy that paired experienced researchers across multiple sites with trainees, ensuring continuity despite funding fluctuations.

The consortium's longevity stems from its adaptive funding portfolio that combines core infrastructure support with project-specific grants. The network coordinator at Uganda Virus Research Institute explains: “We learned early that depending on single large grants made us vulnerable. Instead, we developed a diversified approach where smaller, focused studies maintain our operations while we pursue larger opportunities”.^7^

During the critical 2020 to 2023 period, when many research programs faced 40–60% funding cuts, the EACCR demonstrated exceptional adaptability. They rapidly pivoted to COVID-19 research, leveraging established infrastructure and protocols to conduct urgent studies on vaccine effectiveness, treatment outcomes, and public health interventions across six countries simultaneously. This flexibility not only maintained operations but secured additional emergency funding worth €8.5 million from various sources.^8^

The consortium's HIV and tuberculosis research exemplifies collaborative efficiency. Rather than each site conducting independent trials, they coordinate multi-site studies, providing robust statistical power while sharing costs. Their landmark HIV prevention study included over 12,000 participants across six countries, achieved at 50% lower cost per participant than comparable single-country studies, while generating policy-influencing results adopted by national health ministries.^9^

### Case Study 2: Makerere University's Climate-Health Nexus Program

James Kasaija at Makerere University's School of Public Health faced a devastating 65% budget cut to his climate and health research program in 2022. Rather than abandoning ambitious research objectives, his team revolutionized their approach through community-based participatory research requiring minimal equipment but generating maximum policy impact.

Their groundbreaking study on climate change impacts on vector-borne diseases in rural Uganda demonstrates the power of resource-efficient design. Instead of expensive satellite data and complex modeling software costing $50,000 annually, they trained 200 community health workers to collect standardized environmental and health data using smartphone applications. This innovative approach reduced data collection costs by 85% while simultaneously building local research capacity and generating unprecedented community engagement.^10^

The study's findings revealing previously unrecognized altitudinal shifts in malaria transmission patterns directly influenced Uganda's National Vector Control Strategy and attracted follow-up funding worth $1.2 million from three different sources: the Global Fund, a private climate adaptation foundation, and the African Development Bank's climate resilience program.

“We discovered that community members became our most valuable research partners,” notes Kasaija. “Their local knowledge combined with systematic data collection generated insights impossible through traditional top-down approaches, while creating sustainable research infrastructure that continues operating regardless of external funding fluctuations”.^10^

### Case Study 3: The East African Bioinformatics Resource Consortium

Facing the reality that individual universities couldn't afford expensive bioinformatics software licenses or specialized personnel, five East African universities established the East African Bioinformatics Resource Consortium (EABRC) in 2020. This initiative demonstrates how strategic resource pooling can create research infrastructure exceeding what individual institutions could achieve independently.

Catherine Ngugi from the University of Rwanda, who coordinated the consortium's establishment, explains: “Individual institutions were spending $15,000 to 25,000 annually on basic software licenses without accessing advanced capabilities. By pooling resources, we could afford enterprise-level software and hire three full-time bioinformaticians supporting research across all partner universities for less than what individual institutions previously spent on basic licenses”.^11^

The consortium has supported over 300 research projects since its inception, spanning genomic studies of endemic diseases, agricultural biotechnology research, and environmental genomics. Member institutions report 45% increased grant success rates, as proposals now include sophisticated analytical components previously impossible. The shared infrastructure has also enabled collaborative projects that individually weak applications could never achieve.

Most significantly, the consortium has become financially self-sustaining through fee-for-service offerings to government agencies, NGOs, and private companies requiring bioinformatics analysis. By 2024, external service revenue covered 70% of operational costs, with member institutions contributing only minimal amounts for continued access to world-class capabilities.

### Case Study 4: The Rwandan Digital Health Innovation Network

The University of Rwanda's partnership with the government's digital health infrastructure demonstrates how creative partnerships can provide research opportunities impossible through traditional funding. By collaborating with existing digital health systems, researchers gained access to real-time health data and implementation platforms that would cost millions to establish independently.

Jean-Paul Uwimana, who coordinates the program, notes: “We couldn't afford independent digital health research infrastructure, but by partnering with existing government systems, we've conducted implementation science studies influencing policy across multiple health domains. The government benefits from our analytical expertise and evidence generation, while we access data and platforms otherwise impossible to afford”^12^.

Their research on digital maternal health interventions has directly influenced national policy while generating 15 peer-reviewed publications and attracting $800,000 in follow-up funding from international development agencies, impressed by the scalable, sustainable approach.

### Case Study 5: The Kilifi Health and Demographic Surveillance Innovation

The KEMRI-Wellcome Trust Research Programme in Kilifi, Kenya, faced a 55% funding reduction to its demographic surveillance system in 2021. Rather than discontinuing this 25-year longitudinal resource, researchers redesigned data collection using community-based approaches and mobile technology.

Philip Bejon, the program's former director, describes their transformation: “We trained community health volunteers to use tablets for data collection, replacing expensive field teams. Machine learning algorithms now identify data quality issues in real-time, reducing supervision needs by 70%. These innovations reduced operational costs by 50% while maintaining data quality standards that exceed many well-funded international programs”.^13^

This methodology has attracted international attention, with similar surveillance systems across three continents now adopting the Kilifi innovations. The approach has generated new funding partnerships worth $2.1 million while ensuring the surveillance system's sustainability regardless of future funding fluctuations.

### Case Study 6: Busitema University's Agricultural Partnership Model

Busitema University in Uganda created a revolutionary model for sustainable agricultural research through strategic partnerships with farming cooperatives, seed companies, and government extension services. Facing 60% reduced international agricultural research funding, the university redesigned programs to provide direct value to local partners while maintaining scientific rigor.

Moses Tenywa, who leads the program, explains: “We abandoned extractive research models where we studied communities without reciprocal benefits. Our new collaborative partnerships involve farmers and companies co-investing in research directly benefiting them. Our improved cassava varieties program is now 75% funded by seed companies and farmer organizations who profit from innovations we develop together”.^14^

This approach has generated sustainable funding streams totaling $1.4 million annually while ensuring immediate research uptake and impact. The program's success has inspired similar models across East Africa and attracted renewed international interest from donors seeking scalable, sustainable research partnerships.

### Strategic Approaches for Sustainable Research Programs Diversification and Portfolio Management

Successful East African research programs increasingly embrace portfolio approaches combining multiple funding sources, project types, and collaboration models. The most resilient programs maintain 4 to 6 different funding streams, ensuring that reduced support from any single source doesn't threaten overall program viability.

Strategic diversification includes combining core infrastructure grants providing stable operational support, project-specific funding for targeted research questions, service revenue from analytical capabilities offered to external clients, partnership contributions from government agencies, NGOs, and private sector collaborators, and international collaboration funding, accessing diverse geographic funding pools.

### Leveraging Digital Infrastructure and Open Science

East Africa's expanding digital connectivity offers unprecedented opportunities for cost-effective research. Regional mobile phone penetration exceeds 85%, and internet connectivity continues expanding rapidly, enabling innovative data collection and collaboration approaches that dramatically reduce traditional research costs.

The African Population and Health Research Center (APHRC) in Nairobi has pioneered mobile-based data collection platforms, resulting in a 75% reduction in survey costs compared to traditional field-based methods. Their approach combines SMS-based surveys with targeted phone interviews, enabling large-scale data collection with minimal field staff while maintaining data quality standards.^15^

Open science practices offer additional cost-saving opportunities. The University of Dar es Salaam's systematic adoption of open-source statistical software, preprint servers, and collaborative platforms has reduced research publication and analysis costs by 45% while improving research visibility and collaboration opportunities.^16^

### Building Sustainable Capacity and Infrastructure

Investment in local research capacity often provides superior long-term returns compared to expensive equipment purchases or external consultant fees. East African institutions increasingly recognize that developing internal expertise creates sustainable competitive advantages while reducing ongoing operational costs.

The Makerere University-Johns Hopkins University Research Collaboration (MUJHU) demonstrates this approach's potential. Over 15 years, they systematically transferred program leadership from external to local faculty while building comprehensive local capacity in clinical trials management, data analysis, regulatory compliance, and grant writing. By 2023, the program operated entirely under Makerere leadership, with operational costs reduced by 40% while research output and quality increased substantially.^17^

### Regional Integration and South-South Collaboration

East Africa's position within broader African and Global South networks offers unique opportunities for resource sharing and knowledge exchange that transcend traditional North-South funding dependencies. The African Research Universities Alliance (ARUA) and similar regional networks facilitate collaborations that pool resources while addressing shared research priorities.

South-South collaboration often provides more sustainable partnerships than traditional North-South models, as participating institutions face similar resource constraints and institutional contexts. These partnerships frequently generate innovative approaches to common challenges while building regional research capacity and reducing dependency on external funding.

### Policy Engagement and Research Translation

Strategic engagement with policy processes creates opportunities for sustainable research support while ensuring research relevance and immediate impact. East African researchers increasingly recognize that policy engagement generates both funding opportunities and research uptake mechanisms.

The East African Community's Science, Technology, and Innovation Strategy provides frameworks for regional research coordination and funding that researchers can strategically engage. Programs aligning with these regional priorities often access multiple funding sources while contributing to policy development that benefits entire populations.

### Emerging Opportunities and Future Directions

Despite current challenges, several trends suggest reasons for optimism about East African research sustainability. In 2024, 30 endowments will be offered for the Sub-Saharan Africa region for doctorate (€10,000) and post-doctorate (€15,000) opportunities, indicating continued investment in regional research capacity.

Climate change research presents particular opportunities, as international climate funding mechanisms increasingly recognize adaptation research in vulnerable regions. East African researchers’ expertise in climate-health interactions, agricultural adaptation, and community resilience positions them advantageously for emerging funding opportunities worth potentially billions of dollars over the coming decade.

Private sector engagement in research continues to expand, particularly in digital health, agricultural technology, and renewable energy sectors. Companies operating in East Africa increasingly recognize local research partnerships for product development and market understanding, creating new collaboration and funding opportunities.

The African Continental Free Trade Area creates new markets for research-based innovations, potentially generating revenue streams that can support continued research activities. Universities and research institutions developing intellectual property and innovation commercialization capabilities may access new funding sources while contributing to regional economic development.

### Quality Maintenance and Ethical Considerations

Maintaining research quality while operating under severe resource constraints requires systematic approaches ensuring methodological soundness without compromising scientific rigor. East African researchers have developed several strategies that maintain scientific integrity while optimizing resource utilization.

The concept of “minimum viable studies” focuses on research designs generating meaningful, publishable results with limited resources. This approach requires careful attention to statistical power calculations, strategic outcome selection, and efficient data collection methods while maintaining ethical standards and scientific validity.^18^

Transparency about limitations becomes crucial when resources are constrained. Clearly acknowledging study limitations given available resources strengthens rather than weakens scientific credibility while contributing to honest scientific discourse about research conducted under resource constraints.

## DISCUSSION

The findings from these six case studies reveal a paradigm shift in how East African researchers approach scientific inquiry under severe resource constraints. This transformation challenges conventional assumptions about the relationship between funding levels and research quality, demonstrating that strategic adaptation can yield more sustainable and impactful research programs than traditional well-funded models.

### Common Success Patterns

Several patterns emerge across successful programs. First, diversified funding portfolios consistently outperformed single-source dependency models, with successful programs maintaining 4–6 different funding streams. This diversification created resilience against individual funding cuts while enabling strategic flexibility in research directions. The EACCR's ability to pivot rapidly to COVID-19 research exemplifies how diversified programs can capitalize on emerging opportunities while maintaining core operations.

Second, community engagement emerged as a critical sustainability factor beyond its obvious ethical imperatives. Programs incorporating genuine community participation, such as Makerere's climate-health initiative and Kilifi's surveillance innovation, achieved dramatic cost reductions while improving data quality and policy relevance. This finding suggests that participatory approaches address multiple challenges simultaneously, creating virtuous cycles of sustainability and impact.

Third, resource pooling through strategic partnerships enabled capabilities impossible for individual institutions. The East African Bioinformatics Resource Consortium demonstrates how collaborative infrastructure development can achieve world-class capabilities at fractional individual costs while creating sustainable revenue streams through service offerings.

### The Innovation-Resource Constraint Paradox

Our analysis reveals a counterintuitive relationship between resource constraints and innovation. Rather than simply limiting research capacity, severe funding cuts catalyzed methodological innovations that ultimately strengthened research programs beyond their pre-crisis capabilities. This phenomenon parallels frugal innovation concepts from business literature, where resource constraints drive creative problem-solving that generates sustainable competitive advantages.

Makerere's 85% cost reduction through community-based data collection while generating unprecedented policy impact exemplifies this paradox. The program's forced innovation created a more sustainable, scalable, and impactful research model than traditional approaches. Similarly, Kilifi's surveillance system redesign achieved 50% cost reductions while maintaining superior data quality standards, attracting international adoption and new funding partnerships.

### Sustainability Versus Traditional Metrics

These cases challenge conventional research evaluation metrics that emphasize funding volumes and publication quantities over sustainability and real-world impact. The most successful programs shifted focus toward outcome diversity, including policy influence, capacity building, community benefit, and long-term institutional strengthening alongside traditional academic outputs.

The Rwandan Digital Health Innovation Network exemplifies this shift, generating substantial policy impact and sustainable partnerships while producing fewer traditional publications than comparable programs. However, their research translation and implementation focus created lasting change and attracted sustained funding interest from development agencies prioritizing scalable solutions.

### Regional Context and Global Implications

East Africa's position as a testing ground for sustainable research models offers broader lessons for global research communities. The region's combination of severe resource constraints, urgent research needs, and expanding digital infrastructure creates unique conditions that may preview future research landscapes elsewhere as traditional funding models face increasing pressure.

The success of South-South collaboration models, exemplified by the bioinformatics consortium and EACCR partnerships, suggests alternatives to traditional North-South research dependencies. These partnerships demonstrate how shared challenges can catalyze innovative solutions while building regional capacity and reducing external dependency.

### Limitations and Challenges

Despite these successes, significant challenges remain. Maintaining research quality while operating under resource constraints requires constant vigilance and sophisticated management capabilities that not all institutions possess. The “minimum viable study” framework helps address this challenge but requires careful implementation to avoid compromising scientific rigor.

Additionally, these approaches demand high levels of collaboration and relationship management that can be time-intensive and culturally challenging. Success requires institutional commitment to partnership development and community engagement that may conflict with traditional academic incentive structures prioritizing individual achievement.

The sustainability of these models also depends on continued stakeholder support and favorable policy environments. Economic or political instability could undermine collaborative agreements and community partnerships, highlighting the need for robust risk management strategies.

### Broader Implications for Science Policy

These findings have significant implications for science policy at institutional, national, and international levels. Traditional funding models that emphasize large, individual grants may inadvertently create vulnerability and reduce innovation compared to approaches that encourage collaboration, diversification, and community engagement.

Funding agencies might consider portfolio approaches that combine infrastructure support with project-specific grants while incentivizing inter-institutional collaboration and community engagement. Policy frameworks could also better recognize and reward research translation, capacity building, and sustainable partnership development alongside traditional academic metrics.

At the institutional level, these cases suggest the value of investing in collaboration infrastructure, community engagement capabilities, and diversified funding development rather than focusing exclusively on individual investigator support and equipment acquisition.

### Implementation Strategies and Practical Steps

For researchers and institutions seeking to implement these approaches, several practical steps can initiate transformation toward more sustainable research models:

**Immediate Actions:** The foundation of any successful research transformation begins with a thorough understanding of existing assets and capabilities. Conducting comprehensive audits of current resources, partnerships, and institutional capabilities provides essential baseline information that informs all subsequent strategic decisions. These audits should examine not only financial resources and physical infrastructure but also intellectual assets, staff expertise, existing collaborations, and underutilized capabilities that could be leveraged more effectively.

Identifying potential collaboration partners requires a strategic approach that looks beyond obvious connections to discover organizations with complementary capabilities and shared research interests. This process involves mapping the research landscape to understand who is working on related problems, what unique resources or expertise different institutions possess, and where synergistic opportunities might exist.

Engaging with policy processes and local stakeholders represents a crucial early investment in research relevance and community support. This engagement should begin with listening and learning, understanding the priority concerns of policy makers and community members, and identifying ways that existing or planned research activities could address real-world challenges.

Exploring open science tools and platforms offers immediate opportunities to reduce operational costs while enhancing research capacity and collaboration potential. These tools can streamline data management, facilitate collaboration, improve research reproducibility, and reduce expenses associated with proprietary software and systems.

**Medium-term Developments:** The development of diversified funding portfolios represents a strategic shift from dependency on single sources to a more resilient financial foundation that combines grants, contracts, partnerships, and innovative funding mechanisms. This diversification process requires systematic analysis of funding landscapes to identify opportunities across government agencies, private foundations, industry partners, and international organizations.

Establishing revenue-generating service offerings creates sustainable income streams while simultaneously building institutional research capacity and expertise. These services should leverage existing research strengths to provide value to external clients, whether through consulting, training, testing services, or specialized analyses.

Community engagement strategies must be designed to create genuine partnerships that enhance both research relevance and long-term sustainability. This involves moving beyond traditional outreach models to develop collaborative approaches where communities become active partners in defining research questions, shaping methodologies, and implementing findings.

Building regional and international collaboration networks requires a strategic approach to relationship development that focuses on mutual benefit and resource sharing. This involves identifying institutions and researchers whose capabilities complement existing strengths, developing formal and informal partnership agreements, and creating mechanisms for ongoing collaboration and knowledge exchange.

**Long-term Sustainability:** Developing sophisticated institutional capabilities in grant writing, project management, and partnership development creates the foundational infrastructure necessary for sustained research excellence and growth. Grant writing capabilities must evolve beyond basic proposal preparation to encompass strategic intelligence about funding trends, relationship management with program officers, and the ability to position research within broader scientific and policy contexts.

Innovation and technology transfer capabilities represent a critical investment in translating research discoveries into societal and economic value while generating sustainable revenue streams. These capabilities encompass intellectual property identification and protection, market analysis and commercialization strategies, and the development of industry partnerships that can bring research to scale.

Policy engagement expertise ensures that research activities remain aligned with societal priorities while creating pathways for research uptake and implementation. This expertise involves understanding policy development processes, building relationships with government officials and policy organizations, and developing skills in translating complex research findings into actionable policy recommendations.

Establishing leadership positions in regional research networks and international collaborations creates platforms for shaping research agendas, accessing global expertise, and amplifying institutional impact far beyond what would be possible through isolated efforts. Leadership in these contexts requires demonstrated excellence, diplomatic skills, and the ability to coordinate complex multi-institutional initiatives while maintaining productive relationships across diverse organizational cultures and national boundaries.

## CONCLUSIONS AND RECOMMENDATIONS

Navigating scientific inquiry under severe resource constraints requires fundamental shifts in how East African researchers approach their work, but the experiences documented here demonstrate that such constraints can drive innovation, leading to more sustainable and impactful research programs than traditional, well-funded approaches.

The most successful programs combine strategic collaboration leveraging complementary institutional strengths, innovative methodologies maximizing impact per resource unit invested, diversified funding portfolios reducing dependency on single sources, and robust community engagement ensuring research relevance and sustainability.

Resource limitations, while challenging, often catalyze methodological innovation and collaborative approaches that ultimately strengthen research programs beyond what traditional funding models achieve. East African researchers embracing these approaches position themselves not merely to survive difficult funding environments, but to develop more sustainable, impactful, and community-relevant research programs.

Success requires viewing constraints as creative challenges rather than insurmountable obstacles, building research programs that create value for multiple stakeholders while maintaining scientific rigor. Programs benefiting local communities, policy makers, and international partners while generating high-quality research outputs attract diverse funding sources and achieve long-term sustainability.

As global research funding landscapes continue evolving, East African researchers mastering these approaches may find themselves better positioned than colleagues in traditionally well-funded environments who have not developed similar adaptability, efficiency, and collaboration skills. The future belongs to research programs that demonstrate resilience, innovation, and community relevance regardless of funding fluctuations.

The strategies and case studies presented here offer proven pathways for maintaining scientific excellence while building sustainable research programs that contribute meaningfully to regional development and global scientific knowledge. Implementation requires commitment, creativity, and collaboration, but the potential rewards both for individual researchers and for East African development – justify the necessary investments and adaptations.
